# Supplementary material for: Working dogs in dynamic on-duty environments: The impact of dark adaptation, strobe lighting and acoustic distraction on task performance
Source: PLoS One. 2024 Feb 8;19(2):e0295429. doi: 10.1371/journal.pone.0295429 (PMC10852332; doi:10.1371/journal.pone.0295429)
Supplement: S1 Fig — Outline of the experimental arrangement of the testing room and waiting room. (DOCX) [file pone.0295429.s001.docx]

**SUPPLEMENTAL MATERIALS**

**EXPERIMENTAL ARENA**


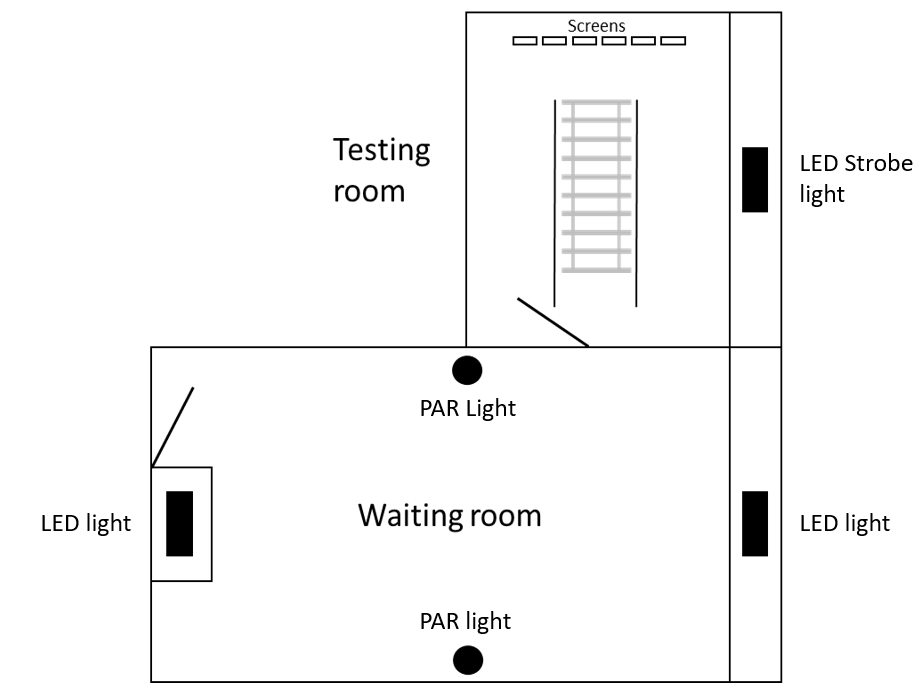


**Supplementary Figure 1.** Outline of the experimental arrangement of the testing room and waiting room.
