# Supplementary material for: Working dogs in dynamic on-duty environments: The impact of dark adaptation, strobe lighting and acoustic distraction on task performance
Source: PLoS One. 2024 Feb 8;19(2):e0295429. doi: 10.1371/journal.pone.0295429 (PMC10852332; doi:10.1371/journal.pone.0295429)
Supplement: S2 Fig — Ater 20 minutes spent in low lighting, dogs were subject to a room illuminated with either a) a white light, b) a red light, or c) a blue light, for one minute before being asked to perform the test task in low lighting. The rooms were lit by two Martin Harman Atomic 3000 LED light and two Ledj Astra 12 quad parabolic aluminized reflector (PAR) lamps. (DOCX) [file pone.0295429.s002.docx]

**SUPPLEMENTAL MATERIALS**

**DARK ADAPTATION CONDITION**


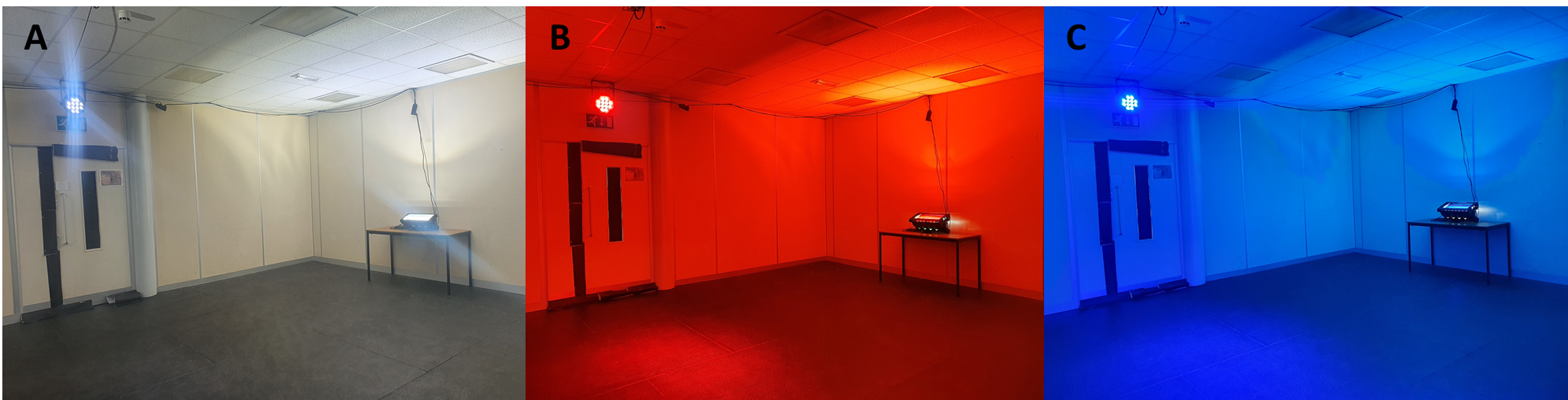


**Supplementary Figure 2.** Dark adaptation condition: after 20 minutes spent in low lighting, dogs were subject to a room illuminated with either a) a white light, b) a red light, or c) a blue light, for one minute before being asked to perform the test task in low lighting. The rooms were lit by two Martin Harman Atomic 3000 LED light and two Ledj Astra 12 quad parabolic aluminized reflector (PAR) lamps.
