# Supplementary material for: Working dogs in dynamic on-duty environments: The impact of dark adaptation, strobe lighting and acoustic distraction on task performance
Source: PLoS One. 2024 Feb 8;19(2):e0295429. doi: 10.1371/journal.pone.0295429 (PMC10852332; doi:10.1371/journal.pone.0295429)

**SUPPLEMENTAL MATERIALS**

**TEST SESSION RECORDING**

Test sessions were recorded by closed-circuit television (CCTV) cameras mounted on the ceiling in strategic locations. All cameras streamed to a HIKVISION digital video recorder which recorded them as separate videos. The cameras were set to capture the following data: Camera 1 (Figure 5, top left) captured an overview of the whole trial, including which screen the dot was presented on; Camera 2 (Figure 5, bottom left) was positioned directly above the dog’s head to allow the coder to determine when the dog was released and when they started the ladder; Camera 3 (Figure 5, top right) was used to determine the moment when the dog entered the goal area, and their choice was recorded; Camera 4 (Figure 5, bottom right) was added to view the majority of the waiting room, including the door to the testing room. An example of a screenshot taken from cameras 1, 2, 3 and 4 (anti-clockwise from the top left).
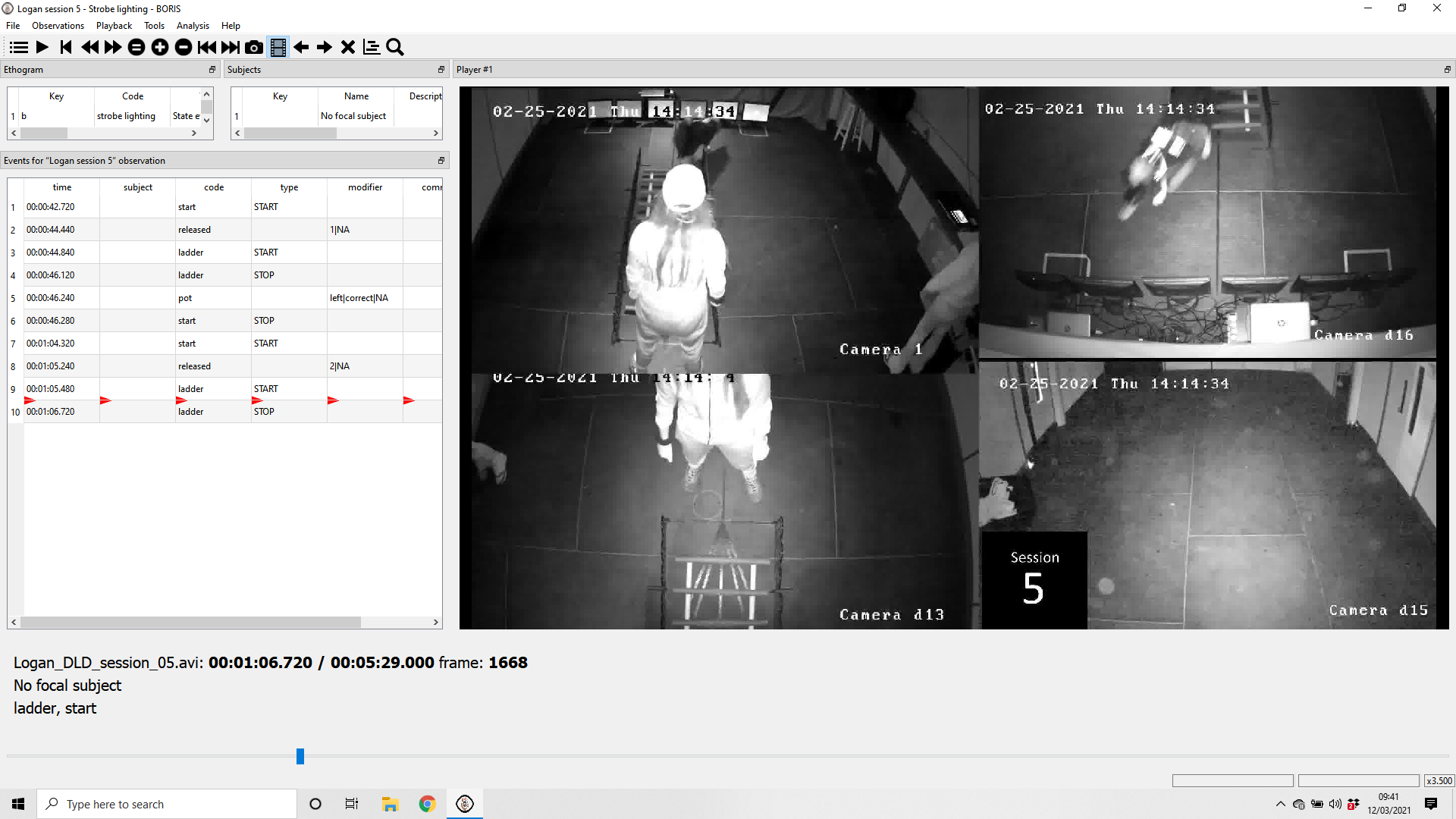

Supplement: S3 Fig — Test sessions were recorded by closed-circuit television (CCTV) cameras mounted on the ceiling in strategic locations. All cameras streamed to a HIKVISION digital video recorder which recorded them as separate videos. The cameras were set to capture the following data: Camera 1 (S3 Fig, top left) captured an overview of the whole trial, including which screen the dot was presented on; Camera 2 (S3 Fig, bottom left) was positioned directly above the dog’s head to allow the coder to determine when the dog was released and when they started the ladder; Camera 3 (S3 Fig, top right) was used to determine the moment when the dog entered the goal area, and their choice was recorded; Camera 4 (S3 Fig, bottom right) was added to view the majority of the waiting room, including the door to the testing room. An example of a screenshot taken from cameras 1, 2, 3 and 4 (anti-clockwise from the top left). (DOCX) [file pone.0295429.s003.docx]
