# Supplementary material for: Working dogs in dynamic on-duty environments: The impact of dark adaptation, strobe lighting and acoustic distraction on task performance
Source: PLoS One. 2024 Feb 8;19(2):e0295429. doi: 10.1371/journal.pone.0295429 (PMC10852332; doi:10.1371/journal.pone.0295429)

**SUPPLEMENTAL MATERIALS**

Canine Cognitive Dysfunction Rating (CCDR) Scale questionnaire used, graphically modified from Salvin et al., (2011).


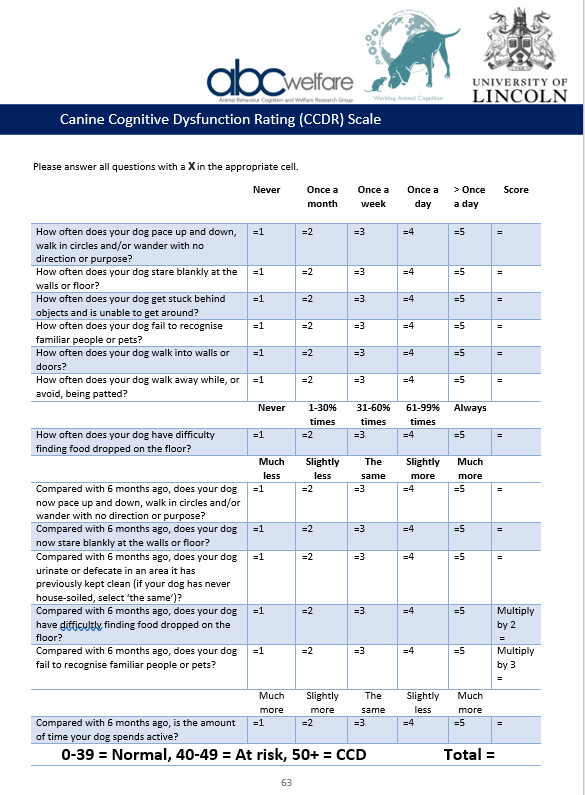


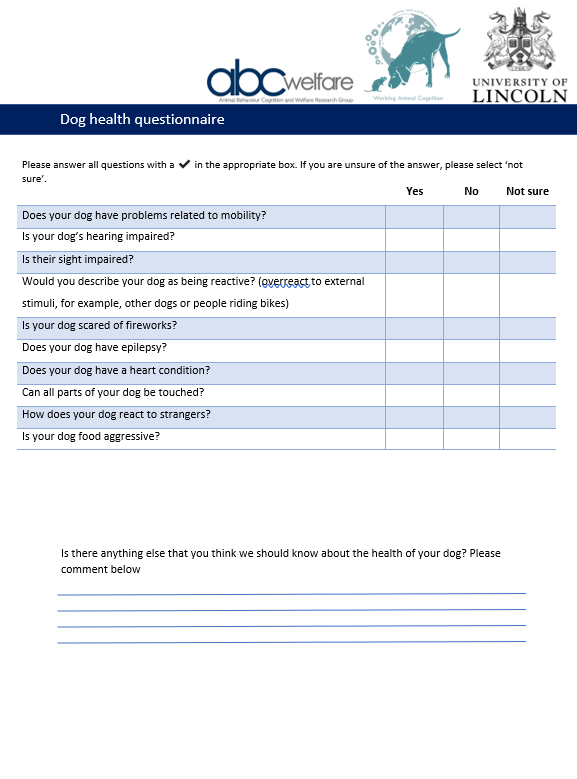

Supplement: S4 Fig — Canine Cognitive Dysfunction Rating (CCDR) Scale questionnaire used, graphically modified from Salvin et al., (2011). (DOCX) [file pone.0295429.s004.docx]
