# Supplementary material for: Working dogs in dynamic on-duty environments: The impact of dark adaptation, strobe lighting and acoustic distraction on task performance
Source: PLoS One. 2024 Feb 8;19(2):e0295429. doi: 10.1371/journal.pone.0295429 (PMC10852332; doi:10.1371/journal.pone.0295429)
Supplement: S1 Table — Twelve pet dogs were recruited for our study. Requirements for recruitment were that dogs i) were older than 1 year, but not older than 10 years, ii) were in good health (including no visual deficits), iii) did not reach the threshold for presence of cognitive dysfunction as assessed using the canine cognitive dysfunction rating scale (Salvin et al., 2011), iv) were comfortable being handled by a stranger, v) were not food aggressive, vi) were at ease in the laboratory environment, and vii) motivated to train. The name, breed, sex, sexual status, age, ear type and eye colour of the dogs who took part in the experiment. ‘GSD’ = German Shepherd, ‘NSDTR’ = Nova Scotia Duck Tolling Retriever, ‘x’ refers to a cross breed. (DOCX) [file pone.0295429.s005.docx]

**SUPPLEMENTAL MATERIALS**

**PARTICIPANT RECRUITMENT**

Twelve pet dogs were recruited for our study. Requirements for recruitment were that dogs:

i) were older than 1 year, but not older than 10 years,

ii) were in good health (including no visual deficits),

iii) did not reach the threshold for presence of cognitive dysfunction as assessed using the canine cognitive dysfunction rating scale (Salvin et al., 2011; Supplementary material 2),

iv) were comfortable being handled by a stranger,

v) were not food aggressive,

vi) were at ease in the laboratory environment, and vii) motivated to train.

**Supplemental Table 1.** The name, breed, sex, sexual status, age, ear type and eye colour of the dogs who took part in the experiment. ‘GSD’ = German Shepherd, ‘NSDTR’ = Nova Scotia Duck Tolling Retriever, ‘x’ refers to a cross breed.

| **Name** | **Breed** | **Sex** | **Sexual status** | **Age (years)** | **Ear type** | **Eye colour** |
| --- | --- | --- | --- | --- | --- | --- |
| Alexis | GSD | Female | Intact | 4 | Pricked | Brown |
| Arlo | Labrador x | Male | Intact | 1 | Folded | Brown |
| Ava | Alaskan Malamute | Female | Neutered | 6 | Pricked | Gold |
| Sky | Kelpie | Female | Neutered | 4 | Pricked | Gold |
| Lyra | Labrador x | Female | Intact | 1.5 | Folded | Brown |
| Poppy | GSD x Malinois | Female | Neutered | 3.5 | Pricked | Brown |
| Hunter | Siberian Husky | Female | Neutered | 6 | Pricked | 1 Blue,  1 Brown |
| Hector | NSDTR | Male | Neutered | 5 | Folded | Gold |
| Lilly | Border Collie | Female | Intact | 1.5 | Rose | Brown |
| Logan | Border collie | Male | Intact | 3 | Pricked | Brown |
| Max | Welsh Springer Spaniel | Male | Intact | 1 | Lop | Brown |
| Pan | Siberian Husky | Female | Neutered | 9 | Pricked | Brown |
