# Supplementary material for: Working dogs in dynamic on-duty environments: The impact of dark adaptation, strobe lighting and acoustic distraction on task performance
Source: PLoS One. 2024 Feb 8;19(2):e0295429. doi: 10.1371/journal.pone.0295429 (PMC10852332; doi:10.1371/journal.pone.0295429)
Supplement: S2 Table — The presentation order of red, white and blue light, and 5Hz, 10Hz and 15Hz strobe light frequency. (DOCX) [file pone.0295429.s006.docx]

**SUPPLEMENTAL MATERIALS**

**STIMULUS ORDER PRESENTATION**

**Supplementary Table 2.** The presentation order of red, white and blue light, and 5Hz, 10Hz and 15Hz strobe light frequency.

|  | Colour Light stimuli | | | Strobe Light stimuli | | |
| --- | --- | --- | --- | --- | --- | --- |
| **Order** | **Red** | **White** | **Blue** | **5Hz** | **10 Hz** | **15 Hz** |
| 1st | 3 | 3 | 4 | 4 | 4 | 4 |
| 2nd | 3 | 3 | 4 | 4 | 4 | 4 |
| 3rd | 4 | 3 | 3 | 4 | 4 | 4 |
