# Supplementary material for: Working dogs in dynamic on-duty environments: The impact of dark adaptation, strobe lighting and acoustic distraction on task performance
Source: PLoS One. 2024 Feb 8;19(2):e0295429. doi: 10.1371/journal.pone.0295429 (PMC10852332; doi:10.1371/journal.pone.0295429)
Supplement: S3 Table — Heart Rate data was recorded continuously during every trial, and was later averaged before, during and after exposure to the stimulus at the point when the dog was released, when it had proceeded halfway down the ladder, and as it approached the goal area. Inter-beat interval data was recorded using a Polar X10 heart rate monitor in conjunction with the HR and HRV Logger app. From this, the dogs’ heart beats per minute (bpm) were calculated across a three second rolling average time frame along with its associated timestamp. This information was then imported into the BORIS software and synchronised with the videos. Heart rate data was averaged across each session. Mean heartrate (beats per minute (bpm)) and standard deviation (±) when dogs were released, started the ladder and approached a goal area in the dark adaptation, strobe lighting and sound condition, on the trial before, during and after a stimulus was presented. (DOCX) [file pone.0295429.s007.docx]

**SUPPLEMENTAL MATERIALS**

**PHYSIOLOGICAL DATA**

Heart Rate data was recorded continuously during every trial, and was later averaged before, during and after exposure to the stimulus at the point when the dog was released, when it had proceeded halfway down the ladder, and as it approached the goal area. Inter-beat interval data was recorded using a Polar X10 heart rate monitor in conjunction with the HR and HRV Logger app. From this, the dogs’ heart beats per minute (bpm) were calculated across a three second rolling average time frame along with its associated timestamp. This information was then imported into the BORIS software and synchronised with the videos. Heart rate data was averaged across each session. Mean heartrate (beats per minute (bpm)) and standard deviation (±) when dogs were released, started the ladder and approached a goal area in the dark adaptation, strobe lighting and sound condition, on the trial before, during and after a stimulus was presented.

|  |  | Release (bpm) | Ladder (bpm) | Goal approach (bpm) |
| --- | --- | --- | --- | --- |
| Dark adaptation | Before | 99.77 ± 24.02 | 100.83 ± 25.17 | 101.70 ± 23.75 |
|  | During | 106.54 ± 20.42 | 106.45 ± 21.44 | 106.99 ± 19.89 |
|  | After | 110.29 ± 23.11 | 110.53 ± 22.94 | 111.00 ± 22.35 |
| Strobe lighting | Before | 106.42 ± 14.07 | 106.19 ± 14.55 | 105.44 ± 15.57 |
|  | During | 107.13 ± 17.88 | 108.44 ± 17.95 | 109.78 ± 16.50 |
|  | After | 107.30 ± 19.94 | 106.84 ± 19.32 | 106.14 ± 17.94 |
| Sound | Before | 114.12 ± 25.60 | 115.18 ± 31.17 | 110.17 ± 20.43 |
|  | During | 113.41 ± 47.99 | 102.96 ± 18.89 | 117.65 ± 28.83 |
|  | After | 104.12 ± 18.84 | 101.34 ± 20.11 | 101.01 ± 20.51 |
